# Identification and characterization of novel cecropins from the *Oxysternon conspicillatum* neotropic dung beetle

**DOI:** 10.1371/journal.pone.0187914

**Published:** 2017-11-29

**Authors:** Lily Johanna Toro Segovia, Germán Alberto Téllez Ramírez, Diana Carolina Henao Arias, Juan David Rivera Duran, Juan Pablo Bedoya, Jhon Carlos Castaño Osorio

**Affiliations:** Center of Biomedical Research. Group of Molecular Immunology. Universidad del Quindío, Armenia, Quindío–Colombia; Centro Nacional de Biotecnologia, SPAIN

## Abstract

Dung beetles are exposed to a complex microbiological ecosystem during their life cycle. Characterization of novel host-defense peptides (HDP) is essential to understanding the host innate immune response in insects. It constitutes a promising alternative to look for new therapeutic agents against pathogenic microbes. We identified four new HDP, Oxysterlins 1, 2, 3, and 4 from the transcriptome of the *Oxysternon conspicillatum* dung beetle. These HDP display a highly conserved signal peptide and a mature peptide, characterized by an overall positive charge (cationic) (pI: 10.23–11.49), a hydrophobic ratio (ΦH: 35–41), and amphipathicity. Oxysterlins 1, 2, and 3 have a linear α-helix structure, whilst Oxysterlin 4 has a mixture of both α-helix and β-sheet structures without disulfide bonds through bioinformatics prediction and circular dichroism. Oxysterlins are part of the cecropin family group in an exclusive clade related to beetle cecropins. They have predominant antimicrobial activity against Gram-negative bacteria, including multidrug resistant strains (3.12–50 μg/mL) measured by plate microdilution. Their kinetics, in a time-killing curve showed concentration-dependent bactericidal activity. Furthermore, these HDP have low toxicity against human erythrocytes (62.5–500 μg/mL) and Vero cells (250–500 μg/mL). This article describes new HDP of the cecropin family from the *Oxysternon conspicillatum* dung beetle, with antimicrobial activity against multidrug resistant bacteria and low toxicity.

## Introduction

There is an urgent need to search for new antimicrobial agents; indiscriminate use of antibiotic therapy, as well as decreased research and development of new active antibiotics against resistant organisms are leading to a public health crisis [1]. The emergence of resistance to last-resort drugs, like third-generation cephalosporins, is fast outpacing the development of alternative treatments and will influence on the burden of disease, like *E*. *coli* (ESBL and fluoroquinolone-resistant), which is a growing global concern with a reported two-fold increase in all-cause mortality. For *Klebsiella pneumoniae* infections with third-generation cephalosporin resistance, there is a significant increase in bacterium-attributable mortality [2].

Antimicrobial cationic peptides are innate host defense molecules effective against bacteria (Gram-positive, Gram-negative), fungi (yeasts and filamentous), parasites, and–in some cases–enveloped viruses [3]. In insects, a diversity of broad-spectrum antimicrobial peptides are rapidly synthetized and secreted by the fat body and hemocytes upon contact with different microorganisms [4–6]. The genes encoding them are mainly activated in the fat body and hemocytes [7]. Within this diversity of peptides, cecropins are a family of 3–4 kDa, cationic, alpha helix, amphipathic peptides [8–10]. These molecules are devoid of cysteine residues and contain two distinctive helical segments: a strongly basic N-terminal domain and a long hydrophobic C-terminal helix, linked by a short hinge [11].

Invertebrates have become an important target group to look for these effector molecules. Among the Coleoptera family, seven HDP of different families have been isolated from five species, *Zophobas atratus* [12,13]; *Tenebrio molitor* (Cucujoidea) [14–16]; *Holotrichia diomphalia* [17]; *Dichotomus dichotoma* (Scarabaeoidea) [18]; and *Tribolium castaneum* (flour beetle) [19]. Other studies have identified three HDP from *T*. *molitor*; Tenecin 1, 2, and 3 [16,20]. Defensins, Coleoptericins, Cecropins, and antifungal peptides have been isolated and biochemically characterized from beetles [21,22].

*Oxysternon conspicillatum* is a neotropic dung beetle with a life cycle where the larvae feed on fungi, decaying organic matter and other organic materials found in dung balls. They are closely exposed to a wide range of microorganisms coevolving with them; therefore, it is likely that *O*. *conspicillatum* may defend itself against invading pathogens by developing strong antimicrobial compounds, among them HDP. Only a few HDP have been identified from a limited number of Coleopteran dung species, which include *Copris tripartitus*, with the CopA3 peptide [23] and *Onthophagus taurus* [24]. Therefore, the purpose of this study was to identify and functionally characterize new HDP from the *O*. *conspicillatum* dung beetle.

## Materials and methods

### Ethics statement

This work was approved by the bioethics committee at Universidad del Quindío (Foundation act 0600, October 29, 2001) under the act number 29 of May 23, 2011. The contract of access to genetic resources was drawn through resolution N° 2073 of Oct 10, 2017.

### Capture and identification of *Oxysternon conspicillatum*

The beetles were captured with pitfall traps in “Pueblo Tapao”, Quindío, at coordinates 4°30’39.0”N and 75°47’12.0”W in decimal format 4.510833–75.786667. The beetles captured were identified as *O*. *conspicillatum*, according to the Weber taxonomic key [25] modified by Medina and Lopera, 2001 [26].

### Induction of immune response and fat body extraction

Induction of the immune response was performed by inoculating 10 μL of *E*. *coli* DH10B at 3 McFarland scale diluted in PBS 1X (NaCl 140 mM, KCl 2.7 mM, NaHPO_4_ 10.1 mM, KH_2_PO_4_ 1.8 mM). The bacteria was inoculated into an adult *O*. *conspicillatum* beetle and, after 18 h, the bacterial challenged specimen was dissected and the fat body extracted and stored at -80°C until use.

### Total RNA extraction, construction, sequencing, and *de novo* assembling of transcriptome

The total RNA from the fat body was extracted by using RNA PureLink^®^ RNA Mini Kit (catalog numbers: 12183018A, Ambion, Life Technologies), according to manufacturer’s instructions. The RNA extracted was quantified by using an Agilent 2100 bio-analyzer for quality and quantified with an RNA integration number (RIN). The RNA library was prepared by using Illumina^®^ TruSeq™ RNA Sample Preparation Kit and the transcriptome was sequenced in a HiSeq 2000 sequencer (Illumina, San Diego, CA, USA) by using paired-end sequencing with 100 bp read length. *De novo* reconstruction of transcriptome from RNA-seq data was constructed with the Trinity platform [27].

### Oxysterlin identification

To identify HDP from the transcriptome, Blasttx [28] and Blast2GO [29] was performed looking for the Cecropin family domains Pfam: PF00272, Prosite: PDOC00241, UNIPROTKB|P84021, InterPro:IPR000875, Pfam:PF00272, PROSITE:PS00268, EMBL:CM000364, InterPro:IPR020400, ProDom:PD001670, OrthoDB:EOG4F4QV0, EMBL:AB047055, EMBL:AB047056.

### Structure prediction of oxysterlin peptides

For the bio-informatic analysis of oxysterlin peptides, the signal peptide was identified by using SIGNALP [30] (http://www.cbs.dtu.dk/services/SignalP/). The secondary structure was found with PSIPRED [31] (http://bioinf.cs.ucl.ac.uk/psipred/) and JPRED [32] (http://www.compbio.dundee.ac.uk/www-jpred/)) and the tertiary structures were confirmed with RAPTORX (http://raptorx.uchicago.edu/)) [33] and visualized in CHIMERA [34]. The physicochemical characteristics, molecular weight, isoelectric point, amino acid number, and hydrophobic moment were predicted with PROTPARAM [35] (http://web.expasy.org/protparam/) and BIOEDIT [36].

### Circular dichroism of oxysterlin peptides

Circular dichroism (CD) spectra was made to estimate α-helix content percentage and stability. Peptides were prepared in aqueous 30% TFE solution at 100 μM concentration. The CD spectra was measured on a 1-cm-long quartz cell on a Jasco J-810 thermostat spectrophotometer [37]. Circular dichroism spectra resulted from the average of four scans obtained by collecting data at 0.1 nm intervals from 260 to 190 nm at room temperature. The deconvolution analysis was performed in SELCON3, CDSSTR, and CONTINL with SMP56-protein reference set.

### Dendrogram for the cecropins family

To generate a group of sequences, Blastp and tBlasttx were used with the oxysterlins as query and HDP sequences with higher identity were selected. The signal peptide was identified and eliminated; with the group of mature peptide sequences, alignment was achieved by using the Clustal Omega program [38] (http://www.ebi.ac.uk/Tools/msa/clustalw2/) and from this alignment, a phylogenetic tree was generated by using the neighbor-joining method in MEGA 7 [39,40]. The percentage of replicate trees in which the associated taxa clustered together in the bootstrap test (10,000 replicates) was calculated and shown next to the branches [41]. The evolutionary distances were computed through the Poisson correction method [42].

### Chemical synthesis of oxysterlins

The sequences of oxysterlin mature peptides were sent to obtain chemical synthesis by hiring the services of the Company Peptides 2.0, with the following conditions:

Approximately 5 mg were obtained from each oxysterlin 1, 2. 3, and 4 mature peptide; these were synthesized through solid-phase chemical synthesis and purified via RP-HPLC and obtained with a purity of 95.11%, 96.19%, 95.54%, and 95.53%, respectively. The molecular mass was determined through mass spectroscopy.

### Antimicrobial activity assay

Antimicrobial activity of oxysterlins 1, 2, 3, and 4 was tested by the broth microdilution assay, as described with some modifications [43]. The *E*. *coli* (ATCC 35218), *Klebsiella pneumoniae* (ATCC BAA 1705 UPC (+)), *P*. *aeruginosa* (ATCC 105663), and the clinical isolates of *Salmonella typhi*, *E*. *coli (ESBL)*, *Enterobacter cloacae*, *Staphylococcus aureus*, *Staphylococcus saprophyticus*, and *Staphylococcus epidermidis* were used as test bacteria, while *Candida parapsilosis* was used to test antifungal activity.

The bacteria were grown overnight in Mueller Hinton agar (MHA) medium (Scharlau 02-136-500, Barcelona, Spain) and adjusted to an absorbance of 0.4 (A570 nm) (3-5X10^8^ CFU/mL). The bacteria inoculum were adjusted to a final dilution of 1:1000 in M.H (3-5X10^5^ CFU/mL); 90 μL of bacteria, and 10 μL of each peptide were mixed to a final peptide concentration between 200–0.37 μg/mL. The solution was incubated at 37°C for 12 h and resazurin was added to a final concentration of 44 μM (Acros Organics 418900050, Geel, Belgium); the plate was incubated for an additional 2 h and the absorbance values at 570 and 603 nm were measured. A delta value (*i*.*e*., A570—A603) was calculated for each well; the average value of the blanks was subtracted from each sample and the growth percentage was calculated relative to a control. Each concentration was measured in triplicate. The minimum inhibitory concentration (MIC) was defined as the concentration (in μg/mL) that inhibited the visible growth of bacterium [43].

### Time-killing test

The time-killing curves for oxysterlins 1, 2, 3, and 4 were constructed at 0.5, 1, 2, and 4 MICs and tested against *E*. *coli ATCC 35218*. The bacteria were grown and adjusted to an optical density of 0.4 (at 570 nm) (3-5X10^8^ CFU/mL). This inoculum was adjusted to a final dilution of 1:20 (6–10 X10^6^ CFU/mL), 45 μL of this bacterium were mixed with 5 μL of peptide and at different times (0, 30, 60, 120, and 180 min). Resazurin was added to a final concentration of 44 μM. Absorbance was measured at λ1 = 570 nm and λ2 = 600 nm after further incubation (2 h, 37°C) [44].

The CFU/mL-versus-resorufin conversion was obtained from an *E*. *coli* ATCC 35218 control growth curve from the same dilution of the time-killing test; different times from this growth curve were measured through two methods; by plating and counting the CFU/mL in 10 Cm M.H agar plates after incubation for 12 h at 37°C and by adding resazurin at 44 μM and incubating at 37°C for 2 h and reading the A570-603 nm for the conversion of resazurin sodium salt into resorufin. These two parameters were graphed and a regression was performed to find the equation of the resorufin conversion versus CFU/mL. From this equation, the data from the time-killing curve measured in resazurin-to-resorufin conversion (A570-603 nm) was extrapolated in CFU/mL units.

### Cytotoxic activity of oxysterlin peptides

Vero cells (ATCC:CCL-81) were grown in Dulbecco’s Modified Eagle Medium (DMEM)—(Life Technologies 12100–046, New York, USA) supplemented with 10,000 units/ml penicillin and streptomycin, 20 mM L-glutamine and 2% (v/v) Fetal Bovine Serum (FBS) heat inactivated (Eurobio, Les Ulis, France). Cells were cultured in polystyrene 96-well microplates (Costar 3590, New York, USA) at 22,500 cells per well and treated with peptide concentrations ranging from 500 to 20 μg/mL and incubated for 24 h at 37°C and 5% CO_2_ humidity. At the end of this incubation period, resazurin was added to each well at a final concentration of 44 μM and the cells were incubated for 2 h at 37°C with 5% CO_2_. Absorbance was measured at 603 and 570 nm. A delta value was calculated (*i*.*e*., A570—A603) and the average of the controls was subtracted from each well value. Cell viability percentage was calculated in comparison to the untreated control cells [45].

### Human erythrocyte hemolytic activity

Two milliliters of human heparinized blood were centrifuged at 800 g for 10 min at room temperature. The erythrocytes were washed three times with a 1X PBS stock solution (130 mM NaCl, 3 mM KCl, 8 mM Na2HPO4, 1.5 mM K2HPO4, pH 7.4). An erythrocyte dilution at 1:250 was performed from the erythrocyte stock solution and incubated at 37°C for 15 min (work solution). In polypropylene 96-well microplates, the different peptides were added to a final concentration from 1000 to 1.95 μg/mL and 90 μL of the erythrocyte work solution was added to these wells. The erythrocytes were then incubated for 1 h at 37°C and centrifuged at 2000 g for 15 min. The supernatants were taken and the absorbance measured at 540 nm. The absorbance of the hemoglobin from the erythrocytes incubated with 1% (v/v) Triton-X100 was taken as 100% hemolysis control and the hemolysis percentage was calculated as (XiA410—A410 nm blank) *100)/(control Triton A410—A410 nm blank) [46].

### Statistical analysis

All experiments were performed in triplicate and plotted as dispersion graph with the mean value error bars as the standard deviation. To calculate the statistical significant difference, a one-way analysis of variance (ANOVA) was performed with Dunnett`s multiple comparison test with a single-pool variance comparing the treated groups against the control group; a significant critical value of 0.05 was chosen.

## Results

### The *Oxysternon conspicillatum* dung beetle transcriptome had sequences related to the cecropin family

*Oxysternon conspicillatum* adult beetles were inoculated with *E*. *coli* and dissected, as described in the methods section. A total of 200 mg of fat body was extracted per beetle and 10.1 μg of total RNA at 380.1 ng/μL were obtained with an RNA integration number of 7.9 for transcriptome sequencing.

For transcriptome sequencing, a total of 30,787,530 raw reads were obtained by HiSeq 2000 (Illumina) paired-end sequencing with a 101-bp average read length (Sequence read archive: SRP082407). We used the Trinity platform software to perform a paired-end joining *de novo* assembly of the valid reads, 27,603 contigs were obtained with a read length from 201 to 12,138 bp and an N50 of 2,159 bp with version: GEXM00000000.1 and code TSA: GEXM01000001-GEXM01027567 in the NCBI GenBank.

Oxysterlin 1 (contig: comp10536_c0_seq 1; GenBank code: GEXM01019095) was identified with the Pfam PF00272, using the Blast2GO strategy; from this sequence, we performed a stand-alone Blast search with the signal peptide of oxysterlin 1 as query and the assembled transcriptome as database. With the BlastX search, three more cecropins were identified: oxysterlins 2, 3, and 4 (contigs: comp6984_c0_seq 3 (GenBank code: GEXM01014653.1); comp6984_c0_seq 2 (GenBank code: GEXM01014652.1); comp6984_c0_seq 1 (GenBank code: GEXM01014651.1) **(S1 File)**.

### Oxysterlins have a conserved domain and prediction of their physicochemical characteristics

**Fig 1:**
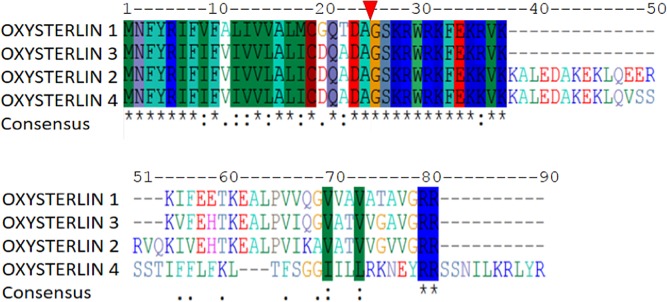
Multiple oxysterlin peptide sequence alignment. The signal peptide was found up to the A23 position. Oxysterlins 1 and 3 have greater similarity. All cecropins have a high identity of the signal peptide and the amine terminal oxysterlin domain of the mature peptide. Oxysterlin 2 had a middle K36-Q54 region that may be the result of alternative splicing.

The open reading frame and the signal peptide of oxysterlin peptides were found for each sequence. Their signal peptide was identified in the A23 amino acid position with high similarity among the four peptides with a consensus sequence MNFRIF(I/V)F(V/A)(I/L)(V/I)V(L/V)AL(I/M)C(D/G)Q(A/T)DA. The mature peptides had an amino terminal consensus sequence (GSKRWRKFEK(K/R)VK) **(Fig 1)**; high isoelectric point and hydrophobic ratio, ranging from 35% to 41% and an α-helix structure, except for oxysterlin 4, which shows a predicted β-sheet and α-helix structure similar to the defensins, but without disulfide bridges and with a high hydrophobic C-terminal domain **(Table 1)**.

**Table 1:** Predicted physicochemical properties of oxysterlin peptides.

Oxysterlin	Mature peptide sequence	aa	M(Da)	ΦH	PI
1	GSKRWRKFEKRVKKIFEETKEALPVVQGVVAVATAVGRR	39	4455.2	41	11.04
2	GSKRWRKFEKKVKKALEDAKEKLQEERVQKIVEHTKEALPVIKAVATVVGVVGRR	55	6327.5	38	10.23
3	GSKRWRKFEKRVKKVFEHTKEALPVIQGVATVVGAVGRR	39	4449.2	38	11.49
4	GSKRWRKFEKKVKKALEDAKEKLQVSSSTIFFLFKLTFSGGIILLRKNEYRRSSNILKRLYR	62	7438.8	35	10.89

aa: amino acids, M (Da): Molar mass in Dalton (Da), ΦH: Hydrophobic ratio, and PI: Isoelectric point.

The oxysterlin tertiary structure prediction was made with RaptorX, by modelling 100% of the mature peptide sequences. The Ramachandran plot of the models showed the percentage of amino acids within permissible regions and angles **(S2 File)**, with 93.7% for oxysterlin 4 and 100% for oxysterlins 2 and 3. The model assessment scores had a good alignment and residues in favored regions, but relatively low scores in the uGDT and p-value; this may be due to the length (<100 residues) of the peptides **(Table 2)**.

**Table 2:** Model assessment of oxysterlin peptide tertiary structure prediction.

Characteristics	Oxysterlin 1	Oxysterlin 2	Oxysterlin 3	Oxysterlin 4
**Template (PDB code)**	1zvuA	5mtvA	1zvuA	3be5A
**Alignment Score**	32	46	29	34
**uGDT (GDT)**	31 (79)	35(64)	30 (77)	32(51)
**P-Value**	5.95x10^-2^	4.45x10^-2^	5.13x10^-2^	2.77x10^-2^
**Residues in most favored regions (Ramachandran plot)**	97.1%	100%	100%	93.7%

**Fig 2:**
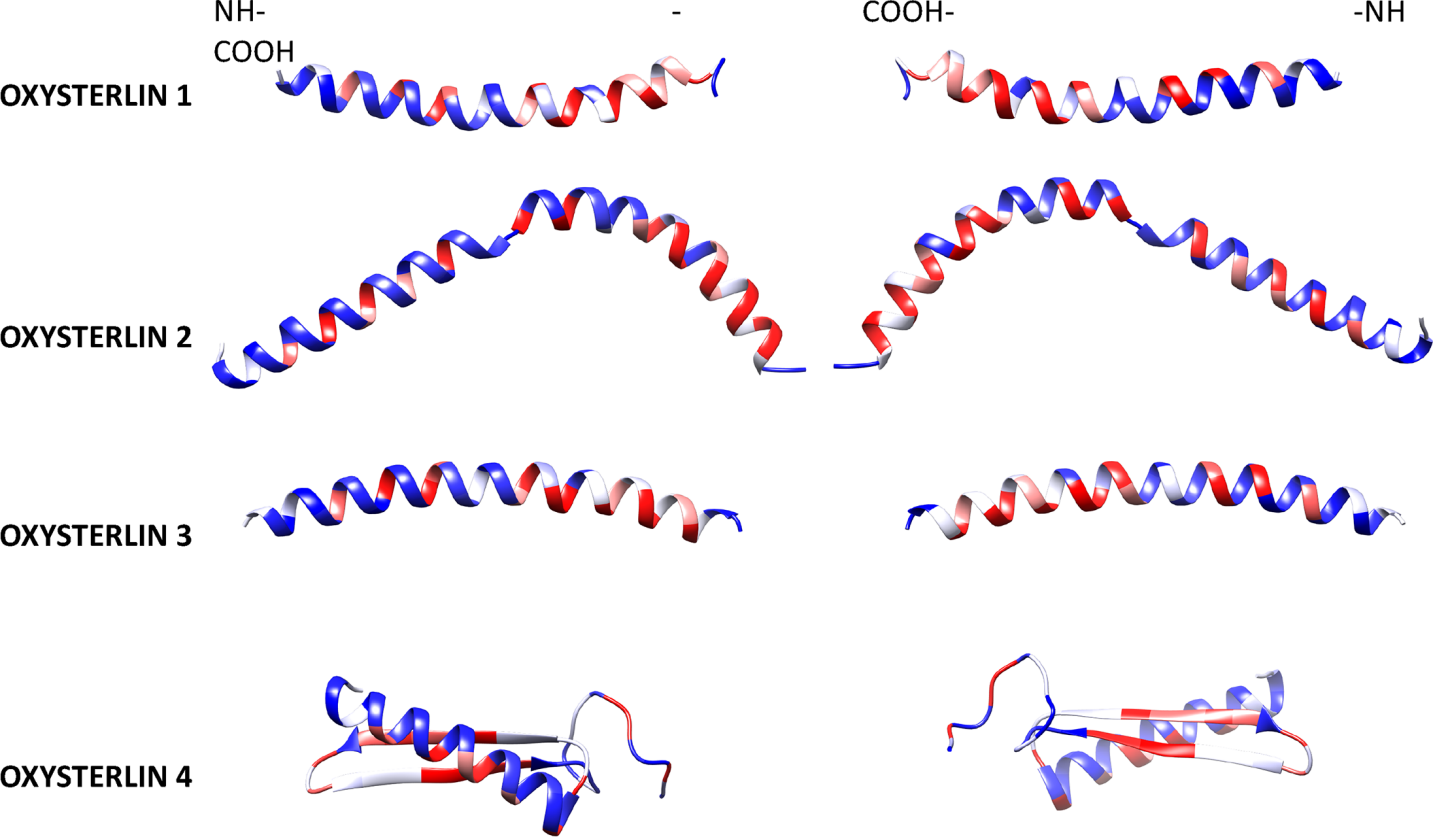
Oxysterlin mature peptide structures. Structures of oxysterlins 1 to 3 had predicted α-helix structure; with an amino terminal region with an amphipathic region corresponding to the oxysterlin domain with a 120° hydrophobic angle and a more hydrophobic carboxyl terminal region. Oxysterlin 4 has an α-helix amino-terminal domain and a β-sheet carboxyl terminal structure. All the structures show an amphipathic amino terminal and a hydrophobic carboxyl-terminal domain (red: hydrophobic, white: neutral, and blue: hydrophilic).

Oxysterlins 1 and 3 showed a continuous α-helix structure. Oxysterlin 2 had two α-helix domains divided by a hinge region in the R27 residue. Oxysterlin 4 shows a predicted β-sheet and α-helix structure without disulfide bonds and with a high hydrophobic C-terminal domain. The hydrophobicity percent and the tertiary predicted structure **(Fig 2)** showed the amphipathic nature of α-helixes, as well as the opposite localization of their hydrophobic and hydrophilic residues through the α-helix structure, with a 120°hydrophobic angle. The tertiary structure was an amphipathic N-terminal helix in which polar/charged residues were segregated on one side of the helix and hydrophobic residues on the other side.

### Circular dichroism

**Fig 3:**
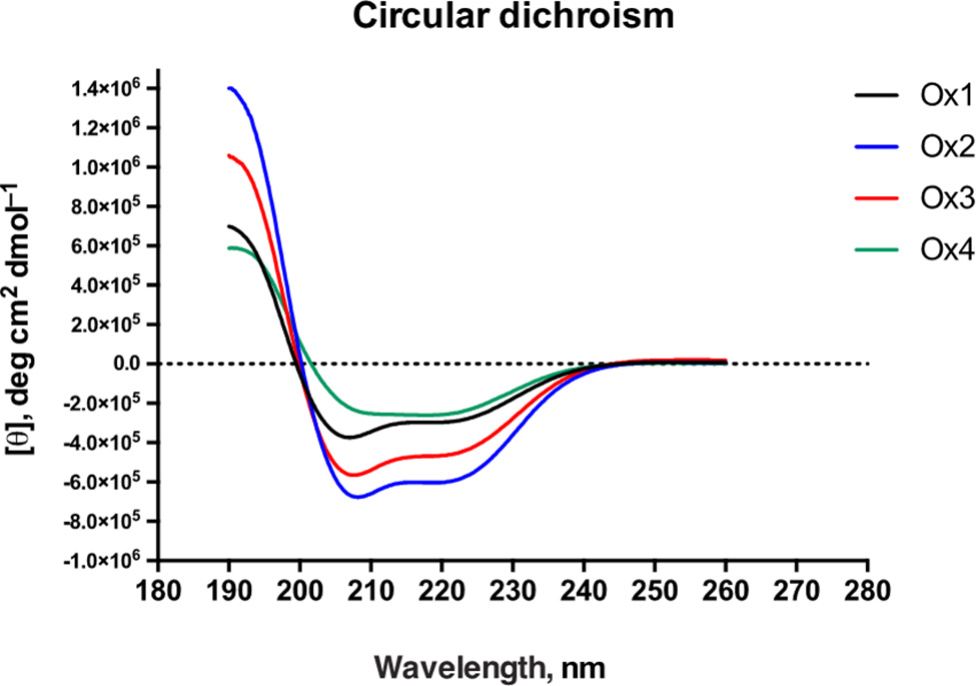
Circular dichroism predicted of oxysterlin peptides. Oxysterlin 1 (black), oxysterlin 2 (blue), oxysterlin 3 (red), and oxysterlin 4 (green). The peptides were dissolved in PBS with 30% TFE; the spectra were made on a JASCO spectrometer. The X axis shows wavelength in nm and the Y axis shows molar ellipticity per residue ([θ], deg cm^2^ dmol^-1^).

The CD spectrum of oxysterlin peptides 1 to 3, dissolved in PBS with 30% TFE, displayed two minimum adsorptions at 208 and 222 nm, which is characteristic of α-helix **(Fig 3 - black, blue, and red lines)** [47]. The CD spectra of oxysterlin 4 showed a significant difference in the positive band at 190 nm **(Fig 3 - green line);** The deconvolution results **(Table 3)** reflect this difference.

**Table 3:** Circular dichroism deconvolution analysis for oxysterlin 4.

Method	H (r)	H (d)	S (r)	S (d)	Turns	Unordered
SELCON3	0.634	0.229	-0.003	-0.001	0.054	0.157
CDSSTR	0.354	0.214	0.072	0.128	0.099	0.141
CONTINL	1.000	0.000	0.000	0.000	0.000	0.000

H(r): α-helix regular. H(d): α-helix distorted. S(r): β-strand regular. S(d): β-strand distorted

### Oxysterlins represent a new diversity of cecropins in beetles

**Fig 4:**
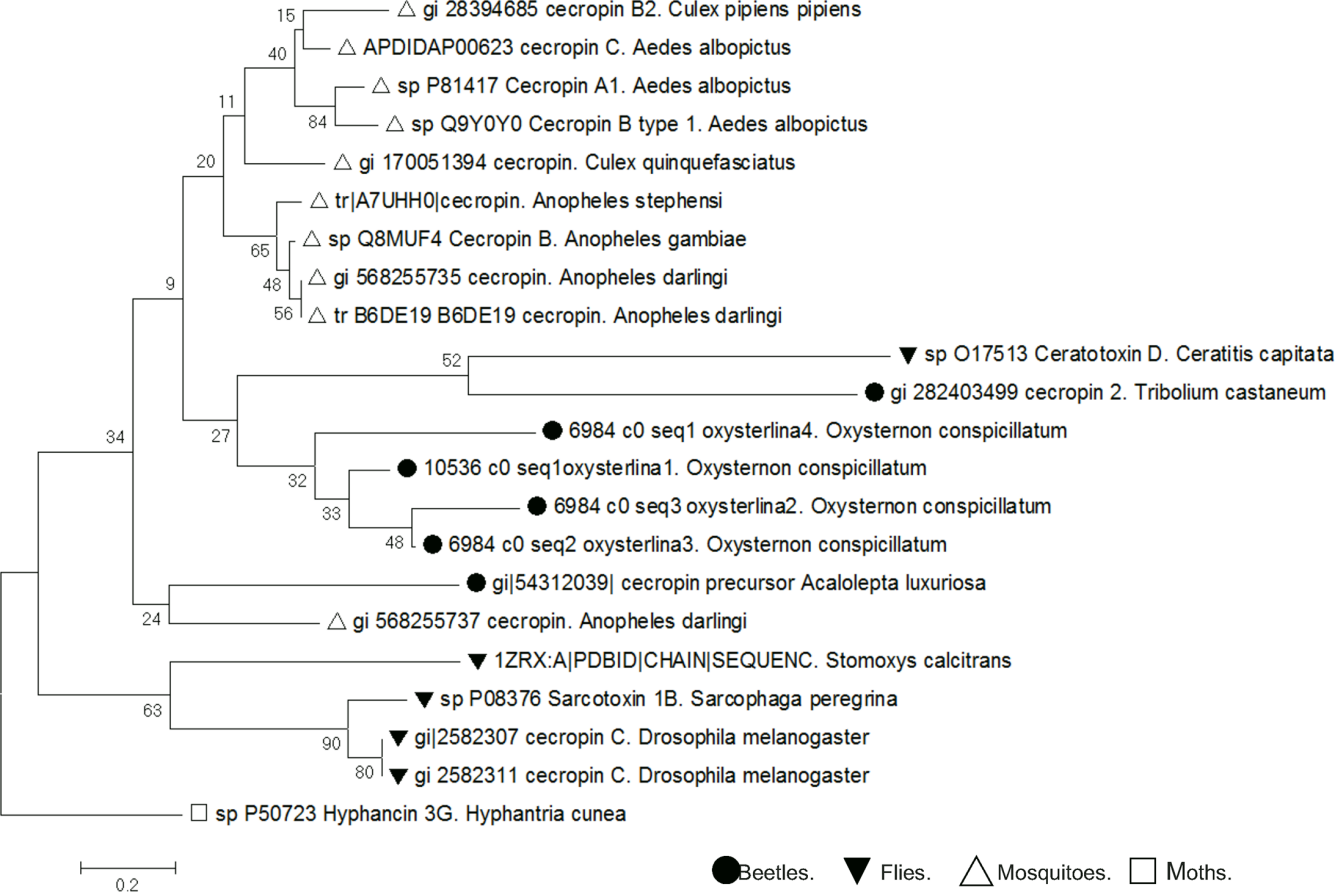
Dendrogram of the cecropin family. The optimal tree with branch length sum = 11.21941379 is shown. The percentage of replicate trees in which associated taxa clustered together in the bootstrap test (10,000 replicates) is shown next to the branches. The tree was drawn to scale, with branch lengths in the same units as those of the evolutionary distances used to infer the dendrogram. The units of the evolutionary distance are the number of amino acid substitutions per site. The analysis involved 21 amino acid sequences. All positions containing gaps and missing data were eliminated. The final dataset had 29 positions.

A cecropin family dendrogram was constructed **(Fig 4).** Cecropins can be divided into three groups: cecropins from Lepidopteran insects belong to the first group, Dipteran cecropins from the second group, and cecropin-2 from coleoptera *Tribolium castaneum*, *Tenebrio molitor*, *Holotrichia sp*, are in the third group. Oxysterlin 3 had 47% identity for cecropin-C from the *Nicrophorus vespilloides* dung beetle (Accession number: XP_017770017.1).

The relation of the aligned sequences shows the *O*. *conspicillatum* peptides in a different root from mosquitoes, flies, and moths; oxysterlins 1 and 3 had the highest similarity amongst them and are more closely related to the *N*. *vespilloides* dung beetle cecropin. This suggests that cecropins occurred in the insects before the divergence of the flies, mosquitoes, and moths. The result also supports that the cecropin molecules have evolved independently among these insect taxa [48].

### The oxysterlins are active antimicrobial peptides against multidrug resistant bacteria

The MIC of oxysterlins against Gram negative, Gram positive, and *Candida parapsilopsis* was measured, as listed in **Table 4**. The antibiotic resistant profile of the bacterial strains was tested previously and the results are listed in **S1 Table**. Oxysterlins 1, 2, and 3 were mostly active against Gram-negative bacteria with MIC ranging from 3.12 to 25 μg/mL. For Gram-positive bacteria, only oxysterlins 1 and 3 were active against *Staphylococcus saprophyticus* with MIC of 50 and 12.5 μg/mL, respectively. Only oxysterlin 1 was active against *C*. *parapsilopsis* and oxysterlin 4 was not active against the microorganisms tested.

**Table 4:** Antimicrobial activity of oxysterlin peptides. Minimum Inhibitory Concentration (MIC) in μg/mL of oxysterlin peptides.

Microorganisms	Oxysterlin 1	Oxysterlin 2	Oxysterlin 3	Oxysterlin 4
*Escherichia coli* ATCC 35218	6.25	50	3.12	>200
*Escherichia coli* clinical isolate (ESBL)	6.25	50	3.12	>200
*Enterobacter cloacae* clinical isolate	12.5	>200	3.12	>200
*Klebsiella pneumoniae* ATCC BAA 1705	>200	25	3.12	>200
*Salmonella typhimurium* clinical isolate	6.25	>200	6.25	>200
*Pseudomonas aeruginosa* ATCC 105663	25	>200	12.5	>200
*Staphylococcus saprophyticus* clinical isolate	50	>200	12.5	>200
*Staphylococcus epidermidis* clinical isolate	>200	>200	>200	>200
*Staphylococcus aureus* clinical isolate	>200	>200	>200	>200
*Candida parapsilopsis* ATCC 22019	50	>200	>200	>200

### The oxysterlins displayed fast-bactericidal activity against *E*. *coli*

We studied the kinetics of the *in-vitro* antibacterial activity exhibited by oxysterlins 1, 2, and 3 through their time-killing curves against *E*. *coli*. Oxysterlin 1 had a MIC of 6.25 μg/mL, oxysterlin 2 (50 μg/mL), and oxysterlin 3 (3.12 μg/mL) against *E*. *coli* ATCC 35218. For these peptides, a concentration-dependent bactericidal activity was found against *E*. *coli* ATCC 35218.

**Fig 5:**
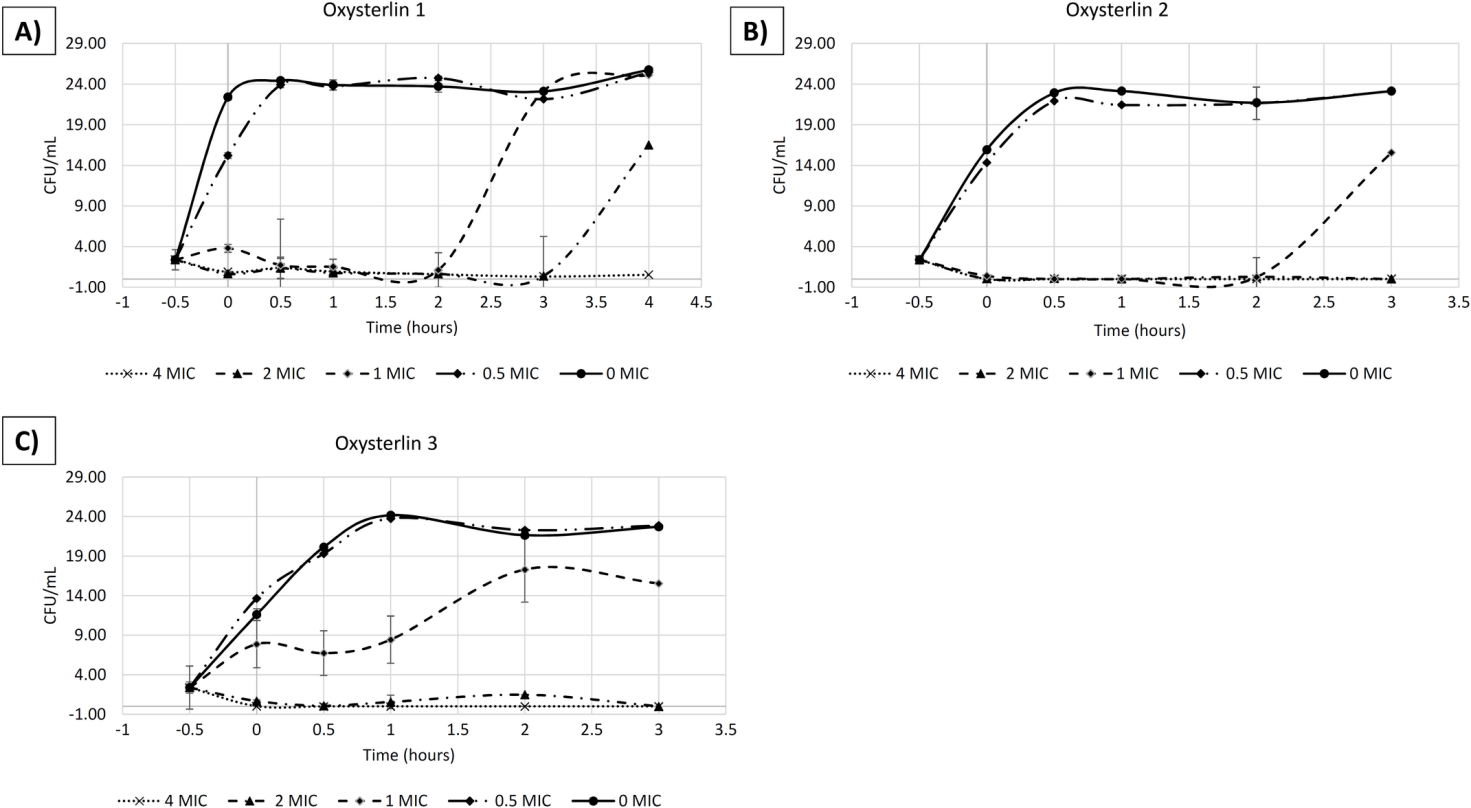
Time-kill curve of oxysterlins 1, 2, and 3 against *E*. *coli* ATCC 35218. (A) Oxysterlin 1, 1X MIC = 6.25 μg/mL. (B) Oxysterlin 2, 1X MIC = 50 μg/mL. (C) Oxysterlin 3, 1X MIC = 3.12 μg/mL.

Oxysterlin 1, from 1 MIC, reduced growth below 1.5 log in CFU/mL during the first 20 min until 120 min of incubation **(Fig 5A)**. Concentrations <0.5 MIC did not inhibit bacterial growth, while concentrations above (2 and 4 MIC) reduced bacterial growth completely during 180 min of the assay, reaching a 2-log reduction. Oxysterlins 2 and 3 partially reduced bacterial growth at 1 MIC with a bacteriostatic-like behavior at this concentration, but at higher concentrations had bactericidal activity with (2 and 4 MIC) with a complete reduction of the CFU/mL at 3 h (**Fig 5B** and **5C**). Bactericidal effect was seen rapidly in the 4 MIC concentration with reduction before 30 min of incubation.

### The oxysterlins had a low toxic profile

**Fig 6:**
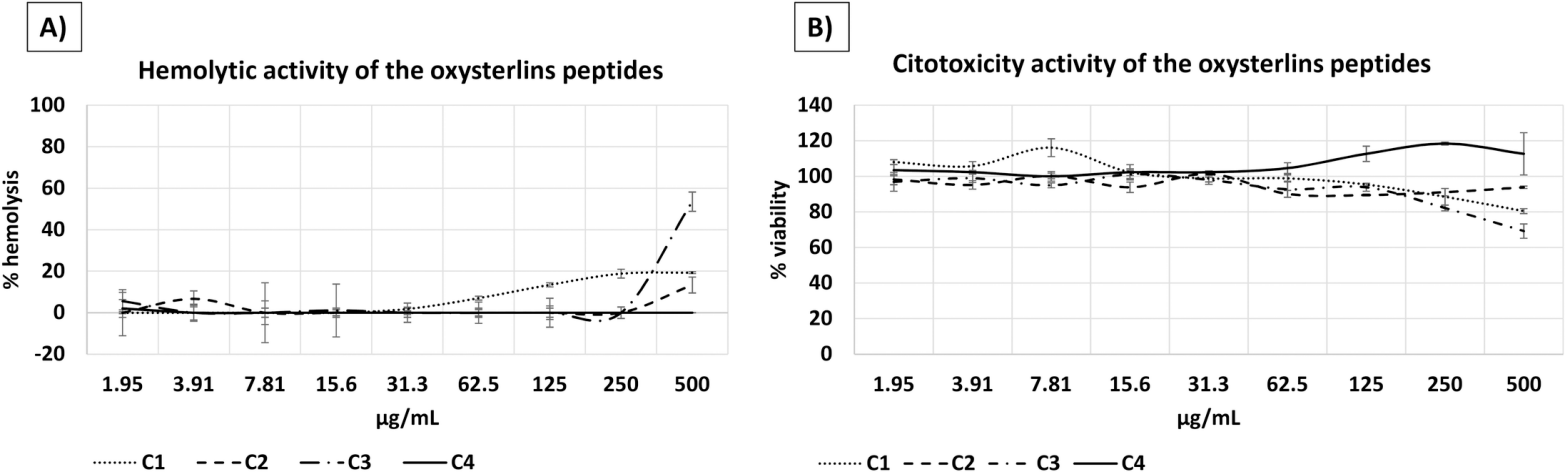
Hemolytic and cytotoxic activity of oxysterlin peptides. **(A)** Hemolytic activity towards human erythrocytes. **(B)** Cytotoxicity activity on Vero cells.

Oxysterlin 1 showed hemolytic activity against human erythrocytes from 500 to 125 μg/mL with 17% to 13% hemolysis, respectively. Oxysterlins 2 and 3 showed hemolytic activity at a concentration of 500 μg/mL with 20% and 50% hemolysis, respectively, while oxysterlin 4 did not show activity against human erythrocytes **(Fig 6A)**.

Cytotoxic activity of oxysterlins 1 and 3 on Vero cells after 24-h incubation showed decreased cell viability over 20% in the highest concentrations (500–250 μg/mL) for both, while oxysterlins 2 and 4 did not show cytotoxicity activity on Vero cells **(Fig 6B)**.

The antibacterial activity of oxysterlin peptides is retained in the conditions of cytotoxic assay (DMEM 1X with 2% Fetal Bovine Serum (FBS); however, the MIC increases four times under these conditions for the three oxysterlins **(Table 5)**.

**Table 5:** MIC of the three oxysterlins under DMEM 1X and 2% FBS conditions. Minimum Inhibitory Concentration in μg/mL of oxysterlin peptides under DMEM 1X and 2% FBS conditions.

Microorganisms	Oxysterlin 1	Oxysterlin 2	Oxysterlin 3
*Escherichia coli* ATCC 35218	25	25	12.5

The therapeutic index (TI) was calculated by dividing the minimum hemolytic concentration by the MIC against *E*. *coli* ATCC 35218; for oxysterlins 1, 2, and 3, the TI were 20, 10, and 160, respectively.

## Discussion

Coleopterans comprise 40% of the 360,000 currently known insect species and are the largest and most diverse order of eukaryotic organisms [49]. Dung beetles (Coleoptera: Scarabaeidae) have a complex life cycle, during which they interact with different microorganisms and environments [50,51]. With over 6,200 species in 267 genera and an estimated 30%–50% of the species still undescribed, dung beetles exhibit diversity comparable to Aves [51]. The *O*. *conspicillatum* dung beetle is a neotropic member of this highly diverse order. Adaptation to their complex life cycle and evolution of their innate immune system in this diverse group of insects makes them an attractive target in the search for new HDP, given that they are the main effector molecules of their immune system. This is corroborated by our work in which we found four new cecropins, three were active against Gram-negative bacteria. By examining their sequence, we can hypothesize that they are the result of genetic duplication and alternative splicing.

Development of high throughput techniques, like next-generation sequencing and transcriptome analysis, is helping to find new antimicrobial peptides in different organisms and coleoptera species. In addition, different strategies of *in silico* analysis are used to predict peptides of therapeutic interest and enrich the search for antimicrobial peptides based on physicochemical properties and nucleotide sequence similarity [52–54]. Nevertheless, the number of host defense peptides in the cecropin family is low, finding mostly defensins and coleoptericins [55]. The new group of cecropins, described in this work, were found by using a gene ontology enrichment strategy based on the blast of the signal peptide of the initial cecropin found by GO against a constructed *O*. *conspicillatum* transcriptome database and bio-informatic validation of the physicochemical properties of the putative sequences (size, net charge, hydrophobic momentum, and structure). With this group of sequences, the *O*. *conspicillatum* cecropin domain was built (GSKRWRKFEK(K/R)VK), corresponding to the amino terminal domain of the mature peptides. The description of this new cecropin domain may help to find new antimicrobial peptides in this highly diverse group of insects.

In insects, cecropins form a large family of cationic α-helical peptides. Few cecropins have been described in dung beetles. This may be explained–in part–because they are a highly diverse superfamily of antimicrobial peptides and a low number exists of available genetic sequences of neotropic beetles; therefore, the homology-based strategy by gene onthology to find new cecropins is limited. This situation was seen in our dendogram where oxysterlins were grouped into a different clade and relatively far from other cecropins, even from the *Tribolium castaneum* beetle, confirming the high diversity of the cecropin superfamily [56]. In spite of the sequence diversity, the physicochemical and structural characteristics of the cecropin superfamily were conserved with a hydrophobic momentum, isoelectric point, amphipathicity, and α-helical structure characteristic of this family, except for oxysterlin 4, which had an α-helical and β-sheet structure similar to the defensins, but without disulfide bridges.

The tertiary structure modelled for the oxysterlins showed the α-helix characteristic of the cecropin family. These models had good scores with respect to the alignment with the template and percentage of amino acids in favored regions, but relatively low scores for global assessment. Nevertheless, these models agreed with the CD experimental data, where oxysterlins 1 to 3 had a characteristic pattern for α-helix, and oxysterlin 4 had a mixture of helix and β-strand.

Cecropins are mainly active against Gram-negative bacteria [8,57]. Oxysterlins 1, 2, and 3, similar to Cecropin A, Aedesin, Lucilin and HKABF [58], were found selective for Gram-negative bacteria and efficiently kill multidrug resistant (MDR) strains, including *E*. *coli* ESBL, *E*. *cloacae*, *Salmonella typhy*, and *E*. *coli* with MIC values between 3.12 and 50 μg/mL. Oxysterlins 2 and 3 were active against *Klebsiella pneumonie* (MIC 3.12 and 25 μg/mL) and oxysterlins 1 and 3 were active against *P*. *aeruginosa* (MIC 25 and 12.5 μg/mL). These MIC are similar to the activity reported for other cecropins, like Cecropin B and Lucilin, ranging from 6.5 to 50 μg/mL [45]. Other cecropins are active against Gram-positive bacteria [59]; in our work, oxysterlins 1 and 3 were active against *Staphylococcus saprophyticus* with MIC of 50 and 12.5 μg/mL, but not against *S*. *aureus* nor *S*. *epidermidis*. Oxysterlin 4 was not active against the microorganisms tested; this finding is likely related to the different sizes, conformational structure, and higher hydrophobicity of the peptide limiting its solubility.

Infections caused by MDR bacteria strains have become a serious global problem and HDP have gained considerable interest as a possible alternative to combat MDR bacteria over the past decade because of their particular action mechanisms and diversity of sequences. The antimicrobial activity of oxysterlins revealed a wide spectrum of activity against pathogenic microorganisms, such as Gram-positive, Gram-negative bacteria and yeasts, like *C*. *parapsilopsis*.

According to the dynamics of oxysterlins 1, 2, and 3 in the time-killing curves, these eliminate *E*. *coli* within less than 20 min at 2 MIC with a concentration-dependent bactericidal effect. This activity is faster than standard antibiotic drugs, like ampicillin, killing bacteria at 2 MIC in 2 h and similar to other antimicrobial peptides [44,60,61]. This concentration-dependent activity may be explained by the mechanisms where reaching a threshold concentration in the membrane is critical for the formation of pores and carpet-like mechanisms that rapidly kill bacteria. Nevertheless, other studies may help to reveal the specific mechanisms of oxysterlin activity.

The therapeutic potential of peptide antibiotic drugs lies in their ability to effectively kill bacterial cells effectively without exhibiting significant cytotoxicity toward mammalian cells. This potential is conveyed by the concept of the relative selectivity index. A high relative selectivity index, thus, incorporates two preferred characteristics of the peptide: high minimum hemolytic or cytotoxic concentration (MHC) and a low Minimum Inhibitory Concentration (MIC) [62]. We found that oxysterlin 2 has a low selectivity index [10]. Oxysterlins 1 and 3 have high selectivity index of 20 and 160, respectively; these indexes could indicate greater specificity of antimicrobial peptides for bacterial cells. Other biophysical and biological properties, like number of hydrophobic interactions, reduced hydrophobicity on the non-polar face, prevented peptide self-association in aqueous conditions, discriminate between eukaryotic and prokaryotic cell membranes. Thereby, this study identified that oxysterlins 2 and 3 may have some desired properties to explore further their potential use against Gram-positive and Gram-negative bacteria.

Oxysterlins 1 and 3, from *O*. *conspicillatum*, have shown the same effects as magainins or cecropins, with cytotoxicity against Vero cells only at higher concentrations, like 500 μg/mL [63]. Some authors suggest that membrane cholesterol in mammalian cells is the base of antimicrobial peptide selectivity [63,64]. However, further studies will be necessary on these effects and their influence on oxysterlin activity.

This study described and determined the antimicrobial capacity of four new HDP, denominated oxysterlins 1, 2, 3, and 4, cecropin-like peptides derived from the fat body of the *O*. *conspicillatum* dung beetle. Their synthetic peptide analogs were evaluated against pathogenic clinical bacteria isolates with MDR profiles, like *E*. *coli* ESBL, *E*. *cloacae*, *Salmonella typhy*, *C*. *albicans*, and *S*. *epidermidis*, providing new insights on the possible use of these molecules to develop new therapeutic alternatives.

## Supporting information

S1 File Oxysterlins genetic sequences. The genetic sequences of the Oxysterlins with the codes reported in the NCBI database.
(DOCX)

S2 File Ramachandran plots of the oxysterlins. Ramachandran plots for the structural models of the Oxysterlins 1 to 4 in the figures A-D.
(DOCX)

S1 Table Antibiotic resistance profile. The antibiotic resistant profile of the bacterial strains.
(DOCX)

## Abbreviations

HDP: (Host Defense Peptides)
ATCC: (American-Type Culture Collection)
ESBL: (Extended-spectrum β-lactamase)
O. conspicillatum: (*Oxysternon conspicillatum*)
MICs: (minimum inhibitory concentrations)
