# Supplementary material for: Identification and characterization of novel cecropins from the Oxysternon conspicillatum neotropic dung beetle
Source: PLoS One. 2017 Nov 29;12(11):e0187914. doi: 10.1371/journal.pone.0187914 (PMC5706684; doi:10.1371/journal.pone.0187914)
Supplement: S1 File — The genetic sequences of the Oxysterlins with the codes reported in the NCBI database. (DOCX) [file pone.0187914.s001.docx]

**S1_File.docx. Oxysterlins genetic sequences.**

OXYS1-

>gb|GEXM01019095.1| TSA: *Oxysternon conspicillatum* comp10536_c0_seq1 transcribed RNA sequence
GTATTATTTATAACACACTTATAAATATAACAATAAATTAGTTATCACAATTAATATTTTTCAGTAGTATTAAAAATATTAGCGACGTCCAACCGCAGTTGCAACTGCCACAACTCCCTGTACAACTGGCAAAGCTTCCTTGGTCTCCTCAAAGATTTTCTTTACACGCTTTTCAAATTTTCTCCACCTTTTAGAACCAGCATCCGTTTGCCCACACATTAAAGCGACGACGATAAGTGCAAATACAAAAATGCGGTAGAAATTCATTTTTACTGTAAAGTAGAGACCGACTATGATATGCACCACAGACAGAT

OXYS2-

>gb|GEXM01014653.1| TSA: *Oxysternon conspicillatum* comp6984_c0_seq3 transcribed RNA sequence
ATATTGTCTTATACATACAGGTACGTTATTAATACTGCTTAGTTAAAAAGTCATCGGCGTCCTACAACGCCCACGACGGTTGCAACAGCCTTTATGACCGGTAAAGCCTCTTTAGTATGTTCGACGATCTTCTGTACTCGTTCTTCCTGTAACTTCTCTTTGGCATCTTCTAAGGCCTTCTTTACTTTTTTTTCAAATTTTCTCCATCTCTTAGAGCCTGCATCAGCTTGGTCACAGATTAAGGCAAGGACGACTATCACAAATATGAATATACGGTAGAAATTCATTATTAATATTTACAGTAGCAAAGTAGTAGGATATTAATAACGTTGTATATAAA

OXYS3- >gb|GEXM01014652.1| TSA: *Oxysternon conspicillatum* comp6984_c0_seq2 transcribed RNA sequence
TTTTTTTTACATCTATAAAGATTTATTTAGGGTTTTGCAAGTTACATAATAAATACTTATTATAAAATGTATTACACATATAGGTACAATATTAATACTGCTTATTGTTAGTTGAAAAGTCATCGGCGTCCTACAGCACCCACAACGGTTGCAACACCCTGTATAACCGGTAAAGCCTCTTTGGTGTGTTCGAAGACCTTCTTTACTCGTTTTTCAAATTTTCTCCATCTCTTAGAGCCTGCATCAGCTTGGTCACAGATTAAGGCAAGGACGACTATCACAAATATGAATATACGGTAGAAATTCATTATTAATATTTACAGTAGCAAAGTAGTAGGATATTAATAACGTTGTATATAAA

OXYS4->gb|GEXM01014651.1| TSA: *Oxysternon conspicillatum* comp6984_c0_seq1 transcribed RNA sequence
ATATTGTCTTATACATACAGGTACGTTATTAATACTGCTTAGTTAAAAAGTCATCGGCGTCCTACAACGCCCACGACGGTTGCAACAGCCTTTATGACCGGTAAAGCCTCTTTAGTATGTTCGACGATCTTCTGTACTCGTTCTTCCTTAATAAAATAATACCACCAGAAAACGTAAGTTTAAATAAAAAAAAAATAGTACTGCTACTTACCTGTAACTTCTCTTTGGCATCTTCTAAGGCCTTCTTTACTTTTTTTTCAAATTTTCTCCATCTCTTAGAGCCTGCATCAGCTTGGTCACAGATTAAGGCAAGGACGACTATCACAAATATGAATATACGGTAGAAATTCATTATTAATATTTACAGTAGCAAAGTAGTAGGATATTAATAACGTTGTATATAAA.
