# Supplementary material for: Identification and characterization of novel cecropins from the Oxysternon conspicillatum neotropic dung beetle
Source: PLoS One. 2017 Nov 29;12(11):e0187914. doi: 10.1371/journal.pone.0187914 (PMC5706684; doi:10.1371/journal.pone.0187914)
Supplement: S2 File — Ramachandran plots for the structural models of the Oxysterlins 1 to 4 in the figures A-D. (DOCX) [file pone.0187914.s002.docx]

**S2 File.** Ramachandran plots for the structural models of the Oxysterlins 1 to 4 in the figures A-D.

Figure A. Oxysterlin 1

Figure B. Oxysterlin 2

Figure C. Oxysterlin 3

Figure D. Oxysterlin 4
