# Supplementary material for: Identification and characterization of novel cecropins from the Oxysternon conspicillatum neotropic dung beetle
Source: PLoS One. 2017 Nov 29;12(11):e0187914. doi: 10.1371/journal.pone.0187914 (PMC5706684; doi:10.1371/journal.pone.0187914)
Supplement: S1 Table — The antibiotic resistant profile of the bacterial strains. (DOCX) [file pone.0187914.s003.docx]

| **Bacteria** | **Resistance profile** | **Antibiotic** |
| --- | --- | --- |
| *Enterobacter cloacae clinical isolate* | Sensible | Amicacine, Aztreonam, cefepine, cefotaxine, ceftriaxoae, ciprofloxacine, ertapenem, gentamicina, imepenem, levofloxacine, meropenem, pip/tazo, piperaciline, tobramicine, trimet/sulfe. |
|  | Resistant | Amox/Aclav, Ampiciline, cefazolina, cefoxitine, cefuroxime. |
| *Staphylococcus saprophyticus clinical isolate* | Sensible | meticiline, daptomicina <=1 S, Rifampicina <=5 S, trimetroprim-sulfametoxazol <=0,5/9,5 S <=0,5/9,5 S. |
|  | Resistant | Ampicilina, Clindamicina >2 R, Eritromicina > 4 R, Minociclina >8 R >8, oxacilina >2 R 0,5 R,  Penicilina, Rifampicina >2 R, tetraciclina >8 R >8 R, |
| *Staphylococcus aureus* | Sensible | Cefazolina <=2 S, clindamicina <=0,5 S, Daptomicina <=1 S, Eritromicina <=0,5 S, linezolida <= 1 S, minociclina <=1 S, oxacilina <=0,25 S, quinupristina-dalfopristina <= 0,5 S, rifampicina <=0,5 S, vancomicina 1 S. |
|  | Resistant | penicilina G > 1 R, tetraciclina > 8 R, trimetroprim-sulfametoxazol >2/38 R. |

**S3. Table. Appendix 3. Antibiotic resistance profile.**
